# Tumor Cells and Tumor-Associated Macrophages: Secreted Proteins as Potential Targets for Therapy

**DOI:** 10.1155/2011/565187

**Published:** 2011-11-17

**Authors:** Marc Baay, Anja Brouwer, Patrick Pauwels, Marc Peeters, Filip Lardon

**Affiliations:** Center for Oncological Research Antwerp (CORE), University of Antwerp, Universiteitsplein 1, 2610 Wilrijk, Belgium

## Abstract

Inflammatory pathways, meant to defend the organism against infection and injury, as a byproduct, can promote an environment which favors tumor growth and metastasis. Tumor-associated macrophages (TAMs), which constitute a significant part of the tumor-infiltrating immune cells, have been linked to the growth, angiogenesis, and metastasis of a variety of cancers, most likely through polarization of TAMs to the M2 (alternative) phenotype. The interaction between tumor cells and macrophages provides opportunities for therapy. This paper will discuss secreted proteins as targets for intervention.

## 1. Introduction

Inflammatory pathways, meant to defend the organism against infection and injury, as a by-product, can promote an environment which favors tumor growth and metastasis. Several infections, inducing inflammation, have been directly linked to cancer. Well-known examples are *Helicobacter pylori* infection and gastric cancer [1], hepatitis B and C virus and hepatocellular carcinoma [2], and schistosomiasis and bladder cancer [3]. Inflammation has therefore been coined the seventh hallmark of cancer [4–7]. 

Macrophages are among the first cells to infiltrate infected or damaged tissue [8]. Tumor-associated macrophages (TAMs), which constitute a significant part of the tumor-infiltrating immune cells, have been linked to the growth, angiogenesis, and metastasis of a variety of cancers, most likely through polarization of TAM to the M2 (alternative) phenotype. M1 (classical) macrophages are generally characterized by interleukin IL-12^high^, IL-23^high^, and IL-10^low^ phenotype. They produce reactive oxygen and nitrogen intermediates as well as inflammatory cytokines and play a role in Th1 responses. Finally, M1 macrophages mediate resistance against intracellular parasites and tumors. M2 macrophages (characterized by an IL–12^low^, IL-23^low^, IL-10^high^ phenotype) are diverse, but in general are involved in T helper 2 (Th2) response, have an immunoregulatory function, and orchestrate encapsulation and containment of parasites and promote tissue repair, remodeling, and tumor progression. Further subdivision of M2 macrophages into M2a (after exposure to IL-4 or IL-13), M2b (immune complexes in combination with IL-1beta or LPS), and M2c (IL-10, TGFbeta or glucocorticoids) has been suggested [9].

Whereas the vast majority of studies with numerous tumor types, including follicular lymphoma [10], intestinal type gastric cancer [11], pancreatic cancer [12], non-gynecologic leiomyosarcoma [13], and thyroid cancer [14], show that the presence of TAM in the tumor microenvironment is associated with a worse prognosis, some studies claim the opposite [15]. The specific role of TAMs in colon cancer is more controversial, as most studies indicate that peritumoral TAMs prevent tumor development (suggesting polarization of TAMs towards the M1 phenotype); patients with high TAM numbers have better prognosis and survival rate [16–19]. In contrast, intratumoral TAM count has been correlated with depth of invasion, lymph node metastasis, and staging of CRC, suggesting that intratumoral macrophages cause cancer cells to have a more aggressive behavior [20, 21].

These contradictions may be due to differences in tumor biology of different tumor types, but may also be a consequence of markers used for the study of TAM. Frequently, the pan-macrophage/monocyte marker CD68 is used as a marker for TAM, whereas the use of CD163 or CD204 might be more appropriate. In fact, Ohtaki et al. [22] show that whereas presence of CD68+ macrophages was of marginal prognostic significance (*P* = 0.08) in lung adenocarcinoma, the use of CD204 showed a strong association with poor outcome in these patients (*P* = 0.007). Similarly, Espinosa et al. found a very strong association between higher number of CD163+ TAM and myometrial invasion of endometrioid carcinoma. Furthermore, there was a positive correlation between the number of CD163+ TAM in the primary tumor and in regional lymph node metastases [23]. In pancreatic cancer, high numbers of CD163- or CD204-positive macrophages were associated with poor prognosis (*P* = 0.0171); however, this was not the case for the number of CD68-positive macrophages [12].

Finally, regardless of the marker used, it is frequently reported that TAMs are associated with prognosis in univariate analysis, but this association is lost in multivariate analysis [24–26]. An exception to this is Hodgkin's lymphoma, where an increased number of CD68+ macrophages outperformed the international prognostic score in multivariate analysis for disease-specific survival [27].

Nevertheless, it is clear that TAMs play an important role in tumor growth and metastasis. This implies that the interaction between tumor cells and TAM provides an opportunity for cancer treatment. In this paper, we focus on secreted proteins as targets for intervention.

## 2. Secreted Proteins

### 2.1. CSF-1

The macrophage colony-stimulating factor (CSF-1 or M-CSF) promotes the differentiation and survival of macrophages. The receptor for CSF-1 is a tyrosine kinase receptor encoded by c-fms. Both CSF-1 and the receptor are expressed by tumor cells of different origins [28, 29], and elevated levels are associated with poor prognosis [30–33]. In fact, in epithelial ovarian cancer patients, elevated levels of CSF-1 in serum or ascetic fluid were associated with poor outcome [34], whereas elevated levels after treatment were indicators of recurrence of progression [35].

### 2.2. CCL2

Chemotactic cytokine ligand 2 (CCL2, also known as monocyte chemoattractant protein 1 (MCP1), monocyte chemotactic and activating factor (MCAF), and monocyte secretory protein JE) is produced in a wide range of tumors [36–39]. Expression of CCL2 is correlated with TAM migration to the tumor, with high expression resulting in higher numbers of TAM, as well as a higher growth rate of tumors after *in vivo* transplantation [40]. Beside the effect on monocytes, CCL2 has also been shown to inhibit the generation of tumor-reactive T cells [41]. Furthermore, prognostic analysis revealed that high expression of CCL2 was a significant indicator of early relapse in human breast cancer patients [42], potentially through the expression of angiogenic factors and activation of matrix metalloproteinases [43]. These protumoral effects of CCL2 are in contrast with the findings of Zhang et al. [44], who showed that early recruitment of monocytes, by high-CCL2-producing tumors as opposed to low-CCL2-producing tumors, inhibits tumor growth.

### 2.3. TNF

Whilst TNF-alpha was first identified as a soluble factor capable of inducing tumor necrosis [45], various mechanisms have been described by which TNF-alpha may promote cancer growth, invasion, and metastasis [46]. Two receptors for TNF have been described, TNF-R1 and TNF-R2. TNF-R1 is expressed on all cell types, whereas TNF-R2 expression is limited to endothelial and immune cells [47]. Mice deficient in TNFR1 or TNFR2 were exposed to chemicals to induce skin tumor formation. Tumor multiplicity was significantly reduced in TNFR1−/− and TNFR2−/− mice compared to wild-type mice, suggesting that both receptors have protumor activity. However, TNFR1−/− mice were markedly more resistant to tumor development than TNFR2−/− mice indicating that TNFR1 is the major mediator of TNF-alpha-induced tumor formation [48]. Constitutive production of TNF from the tumor microenvironment is a characteristic of many malignant tumors, and the presence of TNF is often associated with poor prognosis. TNF has been shown to induce tumor cell invasion through NF-*κ*B- and JNK-mediated upregulation of migration-inhibitory factor in macrophages and through enhanced MMP production in tumor cells [49]. TNF further enhances cell migration and metastasis through NF-*κ*B-dependent induction of chemokines, interleukins, and intercellular adhesion molecule-1 [49]. NF-*κ*B, therefore, seems to play a key role in a TNF-induced signaling pathway. NF-*κ*B can be activated by many stimuli, including proinflammatory cytokines (IL-1, TNF), bacteria, LPS, viruses, and cellular stresses (UV, radiation, chemotherapeutics) [50, 51]. Cellular targets of NF-*κ*B are cytokines, including TNF (positive feedback loop, chemokines, adhesion molecules, inducible effector enzymes and regulators of apoptosis, and cell proliferation [51]. Hence, NF-*κ*B plays a central role in inhibition of apoptosis and tumor promotion and progression, suggesting that the use of NF-*κ*B inhibitors might be useful in cancer therapy. Similarly, TNF inhibitors have been used for the treatment of inflammatory and autoimmune diseases, but also for the treatment of cancer. Several drugs are available, including infliximab, a human-mouse chimeric monoclonal antibody, golimumab and adalimumab, fully human monoclonal antibodies, certolizumab pegol, the PEGylated Fab fragment of a humanized monoclonal antibody, and etanercept, a fusion of the TNF receptor and an antibody constant region (Fc). Infliximab [52] and etanercept [53] especially are under study in clinical trials for the treatment of cancer. However, treatment of rheumatoid arthritis and Crohn's disease with anti-TNF drugs, and especially the monoclonal antibodies, was shown to be associated with an increased risk of reactivation of tuberculosis [54]. Therefore, before treatment with anti-TNF antibodies is initiated, a latent tuberculosis infection should be ruled out. Furthermore, in line with the important role of TNF in host defense and tumor growth control, in patients with rheumatoid arthritis treated with anti-TNF antibody therapy, the pooled odds ratio for malignancy was 3.3 (95% confidence interval, 1.2–9.1) and for serious infection was 2.0 (95% confidence interval, 1.3–3.1) [55].

### 2.4. IL-6

Secretion of IL-6 can be induced by exposure of macrophages to LPS, and hence, can be seen as a representative product of the proinflammatory M1-type macrophages. On the other hand, IL-6 promotes cancer cell proliferation while also inhibits apoptosis of cancer cells through activation of signal transducer and activator of transcription 3 (Stat3) [56]. Stat3 is activated by phosphorylation on Tyr-705, which leads to dimer formation, nuclear translocation, and regulation of gene expression. Serine phosphorylation of Stat3, induced by IL-6 stimulation, has been shown to be independent of mitogen-activated protein kinase and sensitive to the Ser/Thr kinase inhibitor H7. PKC delta is likely to be the kinase that phosphorylates Stat3 in response to IL-6 [57, 58]. Additionally, IL-6 acts as an angiogenic factor and has been implicated in many of the same processes as TNF. Notably, during the cross-talk between cancer and inflammatory cells, Stat3 and NF-*κ*B seem to be key transcription factors linking a mutual positive feedback loop and promoting cancer progression [56]. A tissue microarray study on 221 ovarian cancer cases showed that the intensity of IL-6 staining correlated with prognosis [59]. These data provide the rationale for the use of anti-IL-6 antibodies and STAT-3 inhibitors. A number of clinical studies using siltuximab (CNTO 328), a chimaeric anti-IL-6 monoclonal antibody, have been reported [60–63]. Furthermore, a high-affinity fully humanized anti-interleukin 6 monoclonal antibody (mAb 1339) is available and has shown *in vitro *and *in vivo* antimultiple myeloma activity, both alone and in combination with conventional and novel agents against multiple myeloma [64]. Similarly, sirukumab (CNTO 136), a human monoclonal antibody against soluble IL-6, has been investigated in healthy subjects, showing that it is safe and has a low immunogenicity [65]. Finally, a range of STAT3 inhibitors have been tested and shown to have strong growth-inhibitory activity against cancer cell lines *in vitro* and potent antitumor effects *in vivo* (as reviewed by [66]). Currently, two clinical trials are ongoing, evaluating blockade of STAT3 in solid tumors (NCT00696176, phase 0, and NCT00955812, phase 1), but no results are currently available.

### 2.5. CCL5

Chemokine (C-C motif) ligand 5 (CCL5), also known as regulated upon activation, normal T-cell expressed, and Secreted (RANTES), plays an important role in T-cell proliferation and IFN-*γ* and IL-2 production, which promotes the differentiation and proliferation of Th1 cells important for immune defense against intracellular infection. It was shown that the prostaglandin E2, secreted by mammary gland tumor cells, but not by normal mammary gland epithelial cells, inhibited CCL5 expression in macrophages in response to LPS, but not to TNF-*α* stimulation [67]. Furthermore, an inverse correlation between tumoral CCL5 expression and number of macrophages in the tumor microenvironment has been reported [68], which suggests an antitumoral, rather than a protumoral, role of CCL5. However, when an antagonist of the CCL-5 receptors, CCR1 and CCR5, was used in a mouse model of breast cancer, a significant reduction in volume and weight of treated animals versus controls was observed. The antagonist also showed activity against established tumors [69].

### 2.6. CCL18

Chemokine (C-C motif) ligand 18 (CCL18) is a small cytokine belonging to the CC chemokine family. It was identified, more or less simultaneously, from a range of sources, leading to different names: found highly expressed in lung, it was called pulmonary and activation-regulated chemokine [70] (PARC); based on its similarity to CCL3 it was called macrophage inflammatory protein-4 [71] (MIP-4); after being cloned from dendritic cells, it was called dendritic cell-chemokine 1, [72] (DC-CK1); when macrophages were the source for cloning, it was called alternative macrophage activation-associated CC chemokine-1 [73] (AMAC-1). 

CCL18 is predominantly produced by monocytes/macrophages and dendritic cells (DCs). In case of macrophages, expression of CCL18 can be induced both by Th1 signals (i.e., LPS) and by Th2 signals (i.e., IL-4, IL-10, and IL-13). Immunohistochemistry has shown that CCL18 is produced by CD163+ macrophages [74–76]. Immature DCs express high levels of CCL18, but there is controversy on the effect of maturation, with some reports claiming upregulation [77–79] and others claiming downregulation of CCL18 expression [73, 80, 81]. CCL18 is likely to participate in homing of lymphocytes and DC to secondary lymphoid organs. In case of serious inflammation, CCL18 could assist in mounting a primary immune response through the attraction of naïve T cells towards fully matured DCs [82, 83]. However, in the absence of costimulatory molecules, this can lead to the induction of tolerance through the generation of regulatory T cells (Tregs [84–86]). Furthermore, it has recently been shown that CCL18 can convert memory T-cells to Tregs [87]. Tregs, in turn, can upon coculture induce macrophages to display typical features of alternatively activated macrophages such as CD163 and CD206 and increased production of CCL18 [88], providing a positive feedback loop. Finally, as a CCR3 antagonist [89], CCL18 may limit the recruitment of eosinophils and basophils and hence dampen a local pro-allergic reaction [89, 90]. These data on the role of CCL18 under normal physiological conditions gave an indication that CCL18 might play a role in tumor development. This was underscored by the finding of high levels of expression of CCL18 by tumor-associated macrophages in glioma and ovarian and gastric cancer [91–94]. Furthermore, it was shown that the serum level of CCL18 was elevated in epithelial ovarian cancer patients. In fact, in a study of 51 patients with epithelial ovarian cancer, 27 patients with benign ovarian lesions and 29 healthy volunteers, serum CCL18 gave a sensitivity of 84.3% and a specificity of 91.1% [94]. As Duluc et al. [95] showed that IFN gamma was able to switch immunosuppressive TAM into immunostimulatory cells, with a concomitant reduction in CCL18 secretion, this may be a potential route for therapy. 

Recently, PITPNM3 was identified as the functional receptor for CCL18 that mediates CCL18 effect and activates intracellular calcium signaling. This receptor is the mammalian homologue for *Drosophila melanogaster* rdgB, which is an essential protein for photoreceptor-cell survival and light response [96]. However, the protein appears to be also involved in regulation of cytoskeletal elements [96], which may provide a link to invasion and metastasis. In fact, it was shown that suppression of PITPNM3 abrogated the effect of CCL18 on the invasion and metastasis of breast cancer xenografts [97]. This receptor might therefore be a potential target for therapy. 

On the other hand, a tumor-suppressive function of CCL18 cannot be entirely ruled out as Leung et al. [92] reported that in gastric cancer, CCL18 was expressed by a subset of tumor-associated macrophages, located at the tumor invasion front and that high CCL18 expression levels were associated with prolonged overall and disease-free survival.

### 2.7. MMPs

Matrix metalloproteinases (MMPs) are zinc-dependent endopeptidases, which function to degrade all kinds of extracellular matrix proteins. The MMPs have been shown to play important roles in tissue remodeling associated with various physiological and pathological processes such as morphogenesis, angiogenesis, tissue repair, cirrhosis, arthritis, and metastasis. MMP-2 and MMP-9 especially are thought to be important in preparing the way for tumor cells to metastasize. In contrast, MMP-12 seems to have an antitumoral activity, in that it both retards tumor growth and suppresses growth of lung metastases [98]. Similarly, MMP-3 is thought to be expressed as a protective response and may play a role in host defense during tumorigenesis [99], although MMP3 has also been associated to vascular invasion by immunohistochemistry [100]. Furthermore, MMP-3 regulates macrophage secretion of prostaglandin E2 and expression of MMP-9 [101]. Specific endogenous tissue inhibitors of metalloproteinases (TIMPs) act to inhibit MMPs. These TIMPs comprise a family of four protease inhibitors: TIMP1, TIMP2, TIMP3, and TIMP4. It has been shown that in renal cell carcinoma the balance between MMP and TIMP is disturbed, possibly due to the production of radical oxygen species by TAM [102].

## 3. Treatment

### 3.1. Blocking the Differentiation and Recruitment of Macrophages

Although the association between CSF-1 and enhanced tumorigenesis is evident, CSF-1 also plays an important role in lactation, ovulation, preimplantation, and placental function [103, 104], restricting its role as therapeutic target. The expression of c-fms in normal tissue, on the other hand, is limited to macrophages, except during pregnancy [105], making it a better target for therapy, although indiscriminate destruction of macrophages can have serious consequences for health, including decreased liver function and vulnerability to infectious diseases. Nevertheless, a number of agents have been developed to specifically target c-fms, as well as some multitargeted agents, showing c-fms inhibition in enzyme and cell-based assays [106]. Currently, three phase 1 clinical trials involving c-fms inhibitors are recruiting patients (NCT01004861, NCT01316822, and NCT01346358) (clinicaltrials.gov, accessed 2011/08/26). These studies will show whether c-fms inhibitors are of value in cancer therapy or result in unacceptable levels of toxicity.

The minor groove binding agent Yondelis was used to investigate the immunomodulatory effects on leukocytes. At subcytotoxic concentrations, Yondelis inhibited the differentiation of monocytes to macrophages. The production of CCL2 and IL6 by monocytes, macrophages, TAMs, and tumor cells was also markedly reduced [107]. In the case of human myxoid liposarcoma, *in vitro* treatment of primary tumor cultures and/or cell lines with noncytotoxic concentrations of Yondelis selectively inhibited the production of CCL2, IL-6, and VEGF. A xenograft mouse model of human MLS showed marked reduction of CCL2, CD68+-infiltrating macrophages, and CD31+ tumor vessels after treatment with Yondelis. Similar findings were observed in a patient tumor sample excised after several cycles of therapy [108]. 

After subcutaneous injection of prostate cancer cells in male SCID mice, systemic administration of anti-CCL2 antibodies significantly retarded tumor growth and attenuated macrophage infiltration, with a concomitant decrease in microvascular density [109]. Treatment of immunodeficient mice bearing human breast cancer cells with a neutralizing antibody to CCL2 resulted in a significant decrease of macrophage infiltration, angiogenic activity, and tumor growth [68]. Similarly, CCL2 blockade by antimurine CCL2 monoclonal antibodies significantly slowed the growth of primary tumors and inhibited lung metastasis in animal models of non-small-cell lung cancer. The treatment did not have effect on the number of TAM, but seemed to elicit a change of TAM to a more antitumor phenotype [110]. To investigate another route to block CCL2, a dominant negative CCL2 mutant gene was transfected in the thigh muscle in a model of human melanoma cells being implanted onto the back of a mouse. The dominant negative CCL2 inhibited TAM recruitment and partially reduced tumor angiogenesis and tumor growth [111]. 

CNTO 888, a human mAb specific for human CCL-2, is under current investigation in two clinical trials, one as single agent in patients with metastatic prostate cancer (NCT00992186) and the other in combination with standard of care chemotherapy in patients with solid tumors (NCT01204996). Furthermore, MLN1202, a highly specific humanized monoclonal antibody that interacts with CCR2 and inhibits CCL-2 binding, is being used in a phase II trial in patients with bone metastases (NCT01015560). In a related study on MLN1202 treatment in patients at risk of atherosclerotic cardiovascular disease, patients were genotyped for the 2518 A→G polymorphism in the promoter of the MCP-1 gene. Patients with A/G or G/G genotypes in the MCP-1 promoter had significantly greater reductions in high-sensitivity C-reactive protein levels than patients with the wild-type A/A genotype [112]. This polymorphism may also affect the outcome in studies of cancer patients. 

Following the initial report on cyclooxygenase-2 (COX-2) overexpression in colorectal cancer [113], COX-2 has been the focus of attention as a potential target for cancer treatment. In contrast with COX-1, which is constitutively expressed, COX-2 expression levels are low or undetectable in normal tissues under basal conditions, with the exception of the seminal vesicles, kidneys, and certain areas of the brain, and expression levels increase transiently upon stimulation [114]. However, COX-2 overexpression has been found in a wide range of solid and hematological tumors (reviewed in [115]). Clinical and epidemiological investigations as well as experimental studies have shown that COX-2 contributes to tumourigenesis in every stage: tumor initiation, tumor promotion, and tumor spread. One of the mechanisms involved is the creation of an inflammatory environment. As discussed in Section 1, chronic inflammation constitutes a risk factor for carcinogenesis. Prostaglandin E2 (PGE2) is the most abundant among the prostaglandins produced by COX-2-expressing tumors [116]. The release of PGE2 provides a positive feedback loop [117], which ensures lasting levels of COX-2 in the tumor environment. A role of PGE2-dependent signaling pathways has been described in tumor growth, angiogenesis, tumor invasion and metastasis, tumor survival, and tumor immune tolerance (reviewed in [115]). Given the importance of COX-2 and PGE2, a range of COX-2 inhibitors have been developed. These compounds showed encouraging results *in vitro* and *in vivo* [118, 119] and were introduced in clinical trials for both chemoprevention as well as cancer therapy. Three large randomized clinical trials confirmed the efficacy of COX-2 inhibitors for chemoprevention [120–122], however, at the cost of a significant increase in incidence and severity of thrombotic events [123]. This increased risk, however, could not be confirmed in a meta-analysis of 72 studies, unless patients had previous risk factors for cardiovascular disease [124]. 

More specifically, towards a potential association between COX-2 and TAM, the COX inhibitor DFU (5,5-dimethyl-3-(3-fluorophenyl)-4-(4-methylsulphonyl) phenyl-2(5H)-furanone) was investigated in a rat tumor model and significantly reduced the CCL2 production, as measured both in tumor tissue and in the systemic circulation, with concomitant reduction of the tumor size [125]. Despite these, and other encouraging preclinical results, results of large randomized trials, comparing chemotherapy alone or in combination with COX inhibitors, have been less promising so far [126, 127].

### 3.2. Killing of Macrophages in the Tumor Microenvironment

Bisphosphonates are known to kill macrophages. In a study by Gazzaniga et al., clonodrate-loaded liposomes (CLIPs) were administered to melanoma-bearing mice. The macrophage depletion following this treatment resulted in smaller tumors, with fewer vascular structures [128]. Similarly, in an orthotopic, immunocompetent murine model of diffuse malignant peritoneal mesothelioma, intraperitoneal injection of CLIP leads to apoptosis in tumor cells. Furthermore, when CLIP was injected together with mesothelioma cells, there were a 4-fold reduction in number of tumors and a 5-fold reduction in invasion and metastasis, compared to liposome-encapsulated PBS. Even in mice bearing established tumors, i.p. injected CLIP resulted in a significant reduction in number of tumors [129]. In a study investigating the use of CLIP in several types of tumor in mice, Takahashi et al. showed that injection of CLIP in four spots around the tumor on day 0 or 5 after tumor injection and every third day thereafter resulted in tumor rejection after 12 injections. Depletion of macrophages by CLIP injection before radiotherapy increased the antitumor effect of ionizing radiation [130]. The combination of CLIP with the small molecule sorafenib, for the treatment of a mouse metastatic liver cancer model, was shown to inhibit tumor progression, tumor angiogenesis, and the development of lung metastasis significantly better than sorafenib alone [131]. In this study, zoledronic acid, another bisphosphonate, was shown to be even more effective than clodronate [131]. In an *in vitro* model of prostate cancer cell-macrophage interaction, zoledronic acid selectively suppressed the expression of MMP-9 by TAM, whereas the expression of other mediators was not lowered. Zoledronic acid also boosted the production of type-1 cytokines by PC-TAM in response to immunomodulators such as IL-12, which is known to polarize macrophages towards an antitumoral M1 phenotype [132]. In conclusion, depletion of macrophages in and around the tumor has been shown to give encouraging results in mouse models, most likely by taking out the paracrine signaling by TAM to tumor cells. However, in most cases, bisphosphonates were given simultaneously with the challenge with tumor cells, which obviously is not the situation in patients. Nevertheless, bisphosphonates have been used extensively in humans, while it becomes apparent that their usage is not without risk. The most common adverse effects associated with the use of bisphosphonates are renal toxicity, acute-phase reactions, gastrointestinal toxicity, and osteonecrosis of the jaw. The incidence of these adverse events varies significantly among bisphosphonates. Renal toxicity is a potentially life-threatening event reported in studies of zoledronic acid and, to a lesser extent, pamidronate. In contrast, the renal safety profile of intravenous ibandronate and oral bisphosphonates is similar to that of placebo. Acute-phase reactions occur only with intravenous aminobisphosphonates and may be more common with zoledronic acid. Gastrointestinal effects occur only with oral agents (clodronate and ibandronate) [133]. Careful monitoring of patients, not only for the adverse events described above, but also for infectious diseases and liver failure, due to the indiscriminate destruction of all phagocytic myeloid cells by bisphosphonates, is strictly necessary. 

### 3.3. Repolarization of TAMs

Administration of the proton pump inhibitor pantoprazole to mice with T-cell lymphoma resulted in enhanced TAM recruitment to the tumor environment. These TAMs had the M1 phenotype. Pantoprazole leads to a reversal of immunosuppression and a shift in the cytokine profile [134]. The antitumor effect of pantoprazole was evaluated *in vivo* by a xenograft model of nude mice. After pantoprazole treatment, apoptotic cell death was seen selectively in cancer cells. By contrast, normal gastric mucosal cells showed resistance to pantoprazole-induced apoptosis through the overexpression of antiapoptotic regulators including HSP70 and HSP27 [135]. A phase I study evaluating pantoprazole in combination with doxorubicin for advanced cancer patients is currently recruiting patients (NCT01163903).

IL12, which promotes tumoricidal responses and is normally produced by M1 macrophages, induces tumor regression when used in tumor-bearing mice [136]. This treatment induced a reduction of M2-associated chemokines and an increase in M1-associated chemokines [136]. Further study by this group revealed that the rapid release of IL-15 after IL-12 treatment is essential for infiltration of the tumor and surrounding tissue by leukocytes, including CD8+ T cells, substantiating the repolarization by IL-12 to M1 [137]. In a study by Airoldi et al., the IL-12 receptor beta2 unit was introduced into Calu6 cells by transfection. IL-12 treatment of transfected Calu6/beta2(+) cells inhibited angiogenesis *in vitro*. Tumors in SCID/NOD mice, formed by cells transfected with IL-12Rbeta2, were significantly smaller following IL-12 versus PBS treatment due to inhibition of angiogenesis and of IL-6 and VEGF-C production [138]. Application of repeated doses of IL-12 to cancer patients resulted in a Th1 to Th2 shift (increase in IL10, decrease in IFN-gamma, TNF-alpha, and IP10 in serum of the patients) [139, 140]. This may indicate a potential limitation of the use of IL-12 as a single agent, which is underscored by the finding of a limited efficacy in most clinical trials with IL-12 [141]. However, combined administration of IL-12 with other cytokines, such as IL-2, IL-15, IL-7, IL-21, IL-18, GM-CSF, or IFN-alpha, seems to overcome this problem [142]. Furthermore, when coadministered, a lower effective dose of IL-12 is necessary, reducing potential toxicity, as high toxicity is another limitation of IL-12 therapy [143]. Finally, local administration of the cytokine(s), rather than systemic administration, also reduces the problem of toxicity [142]. For polarization of macrophages towards the alternative phenotype (M2), NF-*κ*b needs to be active. When NF-*κ*b signaling is inhibited, the macrophages become cytotoxic to tumor cells, resulting *in vivo* in regression of advanced tumors [144]. Inhibition of NF-*κ*b signaling may therefore be an alternative for IL-12 administration. 

The host-produced histidine-rich glycoprotein (HRG) was shown to inhibit tumor growth and metastasis while improving sensitivity to chemotherapy. This was accomplished by skewing TAM polarization from M2 to M1 phenotype, accompanied by a promotion of antitumor immune responses and vessel normalization, through downregulation of the placental growth factor [145]. The RCAS/TV-A mouse model for gliomas was used to investigate the effect of HRG on brain tumor development. Tumors were induced with platelet-derived growth factor-B (PDGF-B), in the presence or absence of HRG. HRG was found to have little effect on tumor incidence but could significantly inhibit the development of malignant glioma and completely prevent the occurrence of glioblastoma [146].

### 3.4. Inhibition of M2 Macrophage Functions

Prednisolone has been used to investigate its effect on TAM melanoma-bearing mice. The major inhibitory action on tumor growth was the reduction of TAM-mediated production of proangiogenic factors, whereas the production of antiangiogenic factors was hardly affected [147]. Liposomes encapsulating prednisolone phosphate were developed to evaluate the local delivery of liposomal glucocorticoids to the tumor and its importance for the therapeutic response. A single dose of prednisolone liposomes was found to significantly inhibit tumor growth in mice, subcutaneously inoculated with B16F10 melanoma cells. Uptake of liposomes by TAM was limited to only 5% of the TAM population, and the therapy did not lead to TAM depletion. However, a 90% drop in white blood cell count after prednisolone administration was observed. This depletion may reduce tumor infiltration of monocytes, which stimulate angiogenesis, and possibly cocontributes to the antitumor effects [148]. 

Silibinin has demonstrated anticancer effects against, amongst others, human prostate adenocarcinoma cells [149], human ovarian cancer [150], human colon cancer cells [151], and human lung carcinoma cells [152]. Oral silibinin was tested on established lung adenocarcinomas in A/J mice. Silibinin strongly decreased tumor number and size, probably by an antiangiogenic mechanism [153]. One clinical study using silibinin in advanced hepatocellular carcinoma is ongoing (NCT01129570), and another study in men with prostate cancer has been completed [154, 155]. This study showed that high-dose oral silybin-phytosome achieved high blood concentrations transiently, but only low levels of silibinin were seen in prostate tissue. Furthermore, one of the six treated patients developed a grade 4 postoperative thromboembolic event [155]. 

In the FL-2000 trial, patients with follicular lymphoma were randomly assigned to receive standard treatment (cyclophosphamide, doxorubicin, etoposide, prednisolone, and interferon) or standard treatment plus rituximab. This chimeric monoclonal antibody binds to CD20, which is widely expressed on B cells, from early pre-B cells to later in differentiation. In the control arm, a low number of TAM (CD68+) was associated with a better event-free survival, whereas this effect was not observed in the rituximab arm, which suggests that rituximab is able to circumvent the unfavorable outcome associated with a high number of TAM [156]. In fact, after rituximab and cyclophosphamide-doxorubicin-etoposide-prednisone regimen, high TAM content correlated with longer survival rates. In multivariate analyses, TAM content remained an independent prognostic factor for OS and PFS [157]. It was recently shown that, *in vitro*, Ms4a8a mRNA and MS4A8A protein (a CD20 homologue) expression was strongly induced in bone-marrow-derived macrophages by combining M2 mediators (IL-4, glucocorticoids) and tumor-conditioned media [158]. If this CD20 homologue is also expressed on TAM, this could explain the activity of rituximab.

## 4. Conclusions

It is clear that there are several instants of interaction between tumor cells and macrophages where therapeutic intervention is a possibility. On the other hand, early stages of interaction, such as differentiation and chemotaxis, may already have occurred at the time of diagnosis. Whereas in mouse models c-fms inhibitors, anti-CCL2 monoclonal antibodies, or bisphosphonates can be given before, or simultaneously, with inoculation with tumor cells, in humans this is not the case. Limited evidence is available to support posthoc efficacy of these kinds of treatment. 

Perhaps the most interesting intervention would be the repolarization of macrophages, as this will turn the ally into an enemy, fighting the cancer at close range. From the agents described to invoke repolarization, IL-12 might be the most interesting candidate, with 66 clinical trials in different stages of execution. While not designed to investigate the effect of IL-12 on the interaction between tumor and TAM, these studies may reveal positive effects that will pave the way for new studies investigating the effect of IL-12 on TAM, specifically.

Regardless of the route chosen to block interaction between tumor cells and macrophages, it has become clear that whereas a reduction in tumor growth, angiogenesis, and metastasis can be obtained, complete clearance of the tumor is unlikely. Therefore, combination with chemo- and/or radiotherapy will remain essential.
